# Quasi-3D slope stability analysis of waste dump based on double wedge failure

**DOI:** 10.1038/s41598-024-56637-7

**Published:** 2024-03-22

**Authors:** Chong Chen, Huayong Lv, Zhanbo Cheng, Xu Gao, Xinnan Cui, Xingtong Yue

**Affiliations:** 1 Ansteel Beijing Research Institute Co., Ltd, Beijing, 102209 China; 2 School of Architecture and Engineering, Shangqiu Normal University, Shangqiu, 476000 China; 3 School of Engineering, University of Warwick, Coventry, CV47AL UK; 4 Railway Engineering Research Institute, China Academy of Railway Sciences Corporation Limited, Beijing, 100081 China

**Keywords:** Weak foundation, Waste dump, Double wedge failure, Slope stability analysis, Environmental sciences, Natural hazards

## Abstract

The double wedges sliding along the weak layer of the foundation can be observed on the slope of the waste dump and the sliding body is divided into the active wedge and passive wedge by the weak foundation and the failure surfaces of the waste dump. Because the conventional limit equilibrium slice method cannot reflect the polygonal slip surface of the slope of the waste dump with weak foundation, this study proposed a double wedge calculation method for the slope of the waste dump with weak foundation. The limit equilibrium analysis is performed on double wedges by considering the direction and values of the interaction force between double wedges to obtain the safety factor of the slope of the waste dump. Meanwhile, the quasi-3D double wedges stability analysis method of the waste dump slope with weak foundation is proposed by considering the influence of the geometry and sliding direction of the slope surface on the slope stability. The safety factor of the inverted dump slope is 0.82, the volume of the sliding body is 6.43 million m^3^, and the main sliding direction is 20° south by east. The shear strain rate cloud diagram of the section is ‘y’ type distribution, and the sliding body is divided into two independent blocks. The safety factor of the sliding body section obtained by the double wedge method is between 0.76 and 0.92, and the closer to the boundary of the sliding body, the greater the safety factor of the section. The quasi-three-dimensional safety factor obtained by theoretical analysis is 0.817. The results show that the calculation results of quasi-3D double wedge are basically consistent with the calculation results of strength reduction method, while the proposed method is simpler. It can be used as a quick method to evaluate slope stability in engineering practice.

## Introduction

Waste dump normally stores the overburden materials emission from open-pit, which is composed of the upper waste and the lower basement. Especially, the waste can be soil, rock or soil–rock mixture, and the basement can be soil layer or rock stratum. However, it is easy to cause foundation deformation and waste dump landslide if the waste is carried out on the soil and rock layers with weak bearing capacity, such as quaternary topsoil, diluvium or humus^1–6^. Dump is an important part of open-pit mines. Its stability directly affects the safe production and sustainable development of mines. Therefore, scholars at home and abroad have done a lot of research on the stability analysis and evaluation of dump slopes.

Behera et al. analyzed the stability of an opencast coal mine dump in India under different geotechnical parameters and mineralogy^7^. Wang et al. considered the influence of groundwater seepage on the stability of the dump slope and gave the analytical solution of the safety factor^8^. Based on the fluid–solid coupling analysis method, Li et al. analyzed the influence of rainfall on the stability of the dump slope^9^. Rahul et al. used artificial neural network to calculate the safety factor of dump slope, and analyzed the influence of dump slope geometry, geotechnical properties and hydrological conditions on the stability of dump slope^10^. Cho et al.installed wire sensors and rain gauges on the top of the dump slope to study the stability of the dump slope and the natural slope under the dump of a mine in South Korea^11^. Wang et al. revealed the failure process and failure mechanism of the dump and determined the failure mode of the dump^12^. Bharati et al. used the finite element numerical simulation results to analyze the sensitivity of various geometric parameters and strength parameters of the dump slope^13^.

Over the past decade, many open-pit mine dump landslides were caused by weak foundations, such as the dump landslide of Antaibao open-pit coal mine^14^, the large-scale landslide accident of “Southern District” brown coal mine in Greece^15^ and the dump landslide of Miyizhonghe Iron Mine in Panzhihua City^1^.

Soil-based slope stability analysis methods are commonly used for stability evaluation of waste dump slope, mainly including limit equilibrium method and strength reduction method. Moreover, the limit equilibrium method is the most widely used method in engineering practice due to the relatively clear concept and its relatively simple. However, the 2D limit equilibrium method needs to assume the position and shape of the sliding surface, and the force distribution between the vertical strips^16–19^. Meanwhile, the spatial shape of slope surface and the end effect of sliding mass cannot be considered in this method. Generally, the safety factor obtained by 2D limit equilibrium method is conservative. Therefore, it is necessary to perform 3D stability analysis for complex slope conditions. The previous research mainly expanded the 2D slice to 3D strip column for developing 3D stability analysis method. However, it requires more assumptions and the calculation is more complicated. For example, the three-dimensional slope stability analysis method based on finite element stress calculation proposed by Su and Shao must conform to the unified assumption of the sliding direction of the rigid body limit equilibrium method to calculate the three-dimensional sliding direction of the slope^20^. In addition, there is limited difference between the results of 3D calculation and 2D calculation^11,21–23^. Thus, the 3D stability analysis method has not been widely used. In recent years, strength reduction method has rapidly developed and applied because it can not only consider the stress–strain relationship of rock and soil, but also obtain the safety factor of slope and the location of potential slip surface without assuming the position of slip surface in advance^24–27^. Among them, Shen and Karakus proposed a nonlinear SSR technique to analyze the stability of three-dimensional rock slopes^28^.

There are mainly two methods to evaluate 3D stability parameters based on 2D stability calculation. The most risk section is to be selected to perform stability analysis and the obtained safety factor is the evaluation index in 3D slope. However, the method is conservative and it is difficult to accurately determine the position of the riskiest section in practical engineering. On the other hand, multiple groups of sections are selected for a comprehensive evaluation to obtain the safety factor of the 3D slope. Sherard et al. proposed that the safety factor of 3D slope was approximately the ratio of the sum of shear resistance of multi-sliding surfaces to the sum of sliding force^29^. Erikloehr et al. proposed a fast quasi-3D slope stability analysis method through divided the sliding body into strips along the sliding direction and the 2D limit equilibrium method was adopted to evaluate the slope stability of 3D slope^22^. Yu et al. proposed the quasi-3D slope stability analysis method based on limit equilibrium method and strength reduction method by considering the friction of strips. This method can better predict the potential sliding direction and volume^30^.

In summary, many scholars have made many useful achievements in the study of the stability and evaluation of the dump slope. However, the dump slope is not only different from the rock slope, but also different from the general natural soil slope. It is necessary to propose a stability analysis method that conforms to the actual situation of the dump slope. In this paper, a fast calculation method of quasi three-dimensional double wedge stability of dump slope with weak base is proposed by means of theoretical analysis and numerical simulation, which provides a new idea for evaluating the stability of three-dimensional dump slope.

## 2D double wedge stability analysis method

When the double wedge failure occurs in the slope, we can divide the wedge along the sliding surface. The wedge above is the active wedge, and the wedge below is the passive wedge, and the passive wedge is subjected to the oblique downward extrusion of the active wedge.

**Figure 1:**
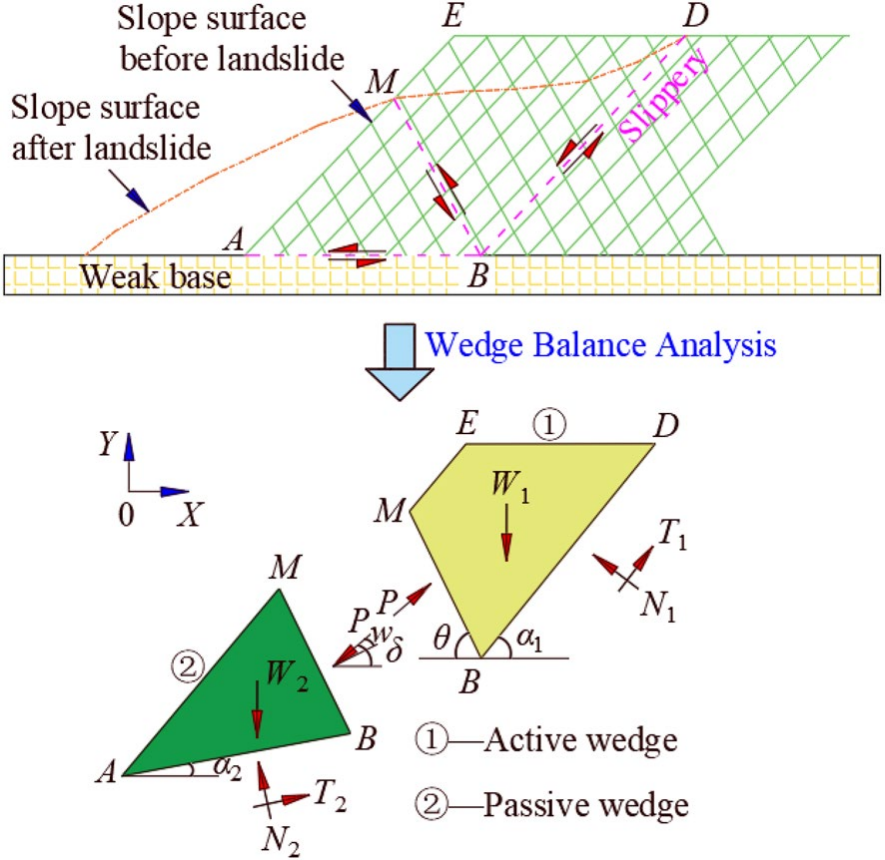
Two-dimensional double wedge stability analysis.

Figure 1 illustrates the double wedges slope stability analysis method to deduce the safety factor of slope through performing balance analysis on the active wedge (①) and the passive wedge (②). The sliding force (*S*_*i*_) and anti-sliding force of active wedge and passive wedge along the sliding surface *BD* and *AB* are as follows, respectively.

1 $$ S_{i} = W_{i} \sin \alpha_{i} $$

2 $$ T_{i} = \frac{{c_{i} L_{i} }}{F} + \frac{{N_{i} \tan \varphi_{i} }}{F} $$

3 $$ N_{i} = W_{i} \cos \alpha_{i} $$

where *i* is 1 or 2 corresponding to active wedge and passive wedge, respectively. *L*_*i*_, *c*_*i*_, *φ*_*i*_ is the length, cohesion, internal friction anlge of the potential sliding surface in the corresponding wedge. *F* is safety factor of double wedges. *α*_*i*_ is the angle between tensional sliding surface *BD* or shear sliding plane *AB* and horizontal plane. *N*_*i*_ and *T*_*i*_ are the normal tangential forces acting on the active wedge by the rear sliding surface or the passive wedge by the basement, respectively. *W*_*i*_ is the gravity of each wedge.

If the active wedge is in the limit equilibrium state, the force *P* providing from sliding surface *BM* to the active wedge can be expressed as follow.

4 $$ \frac{{c_{1} L_{1} }}{F} + \frac{{W_{1} \cos \alpha_{1} \tan \varphi_{1} }}{F} + P\sin \left( {\alpha_{1} - \delta } \right)\frac{{\tan \varphi_{1} }}{F} - W_{1} \sin \alpha_{1} + P\cos \left( {\alpha_{1} - \delta } \right) = 0{\kern 1pt} $$

where *δ* is the angle between the force *P* and the horizontal plane.

Meanwhile, the equilibrium state of passive wedge in sliding surface *AB* is expressed as follow.

5 $$ \frac{{c_{2} L_{2} }}{F} + \frac{{W_{2} \cos \alpha_{2} \tan \varphi_{2} }}{F} + P\sin \left( {\delta - \alpha_{2} } \right)\frac{{\tan \varphi_{2} }}{F} - W_{2} \sin \alpha_{2} - P\cos \left( {\delta - \alpha_{2} } \right) = 0{\kern 1pt} $$

Based on Eqs. (4) and (5), the safety factor (*F*) of slope can be obtained, which is an implicit function. And then the iterative method is applied to solve until the value of two iterations within the allowable error (e.g., 0.001). Under this condition, the double wedges and the whole slide body have the same safety factor.

The angle (*ω*) between the direction of interaction force of double wedges and the vertical direction of interface *BM* can be obtained according to the static equilibrium conditions of the interface *BM* as follow.

6 $$ S_{{\text{m}}} = P\sin \omega $$

7 $$ N_{{\text{m}}} = P{\text{cos}}\omega $$

8 $$ S_{{\text{m}}} = \frac{{c_{1} L_{3} }}{F} + \frac{{N_{{\text{m}}} {\text{tan}}\phi_{1} }}{F} $$

where *S*_m_ and* N*_m_ are the sliding force and vertical force of the interface of double wedges, respectively. *L*_3_ is the length of the potential sliding surface *BM*.

Combined to Eqs. (6), (7) and (8), the following expression can be obtained.

9 $$ P\sin \omega = \frac{{c_{1} L_{3} }}{F} + \frac{{P\cos \omega \tan \varphi_{1} }}{F} $$

10 $$ (F^{2} P^{2} + P^{2} \tan^{2} \varphi_{1} )\sin \omega - 2c_{1} L_{3} P\sin \omega + c_{1}^{2} L_{3}^{2} - P^{2} \tan^{2} \varphi_{1} = 0 $$

Moreover, *ω* and *δ* can meet the following relationship.

11 $$ \omega = \delta + \theta - \frac{\pi }{2} $$

## Quasi-3D double wedges slope stability analysis method

**Figure 2:**
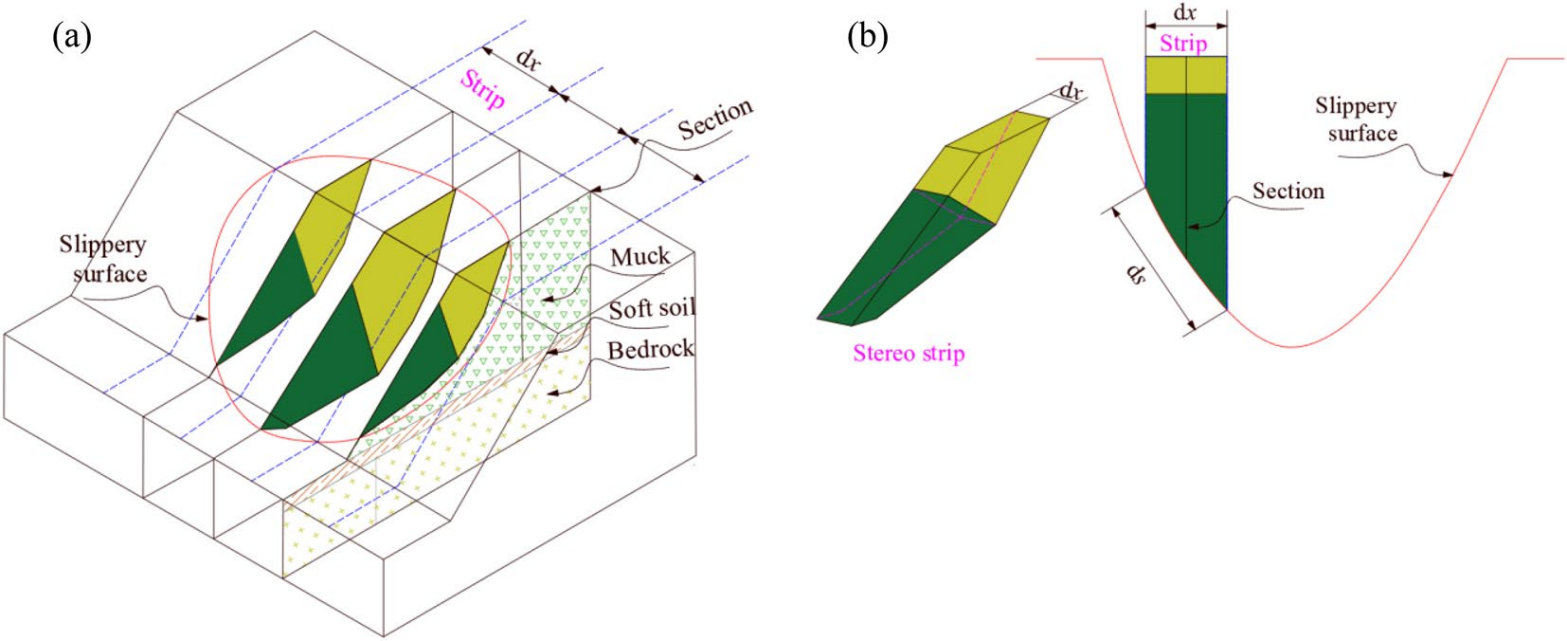
Quasi-3D double wedge stability analysis diagram.

In this study, a quasi-3D double wedges calculation method for waste dump with weak foundation was proposed based on the 2D double wedges stability calculation method. As shown in Fig. 2, the sliding body is divided into strips along the sliding direction and the middle section of each strip is selected for 2D stability analysis. Under this condition, the calculated result of the middle section can be approximately the safety factor of the strip when the width of the strip is small enough. It can be expressed as follow.

12 $$ F_{2} = \frac{{T_{{\text{t}}} }}{{S_{{\text{t}}} }} $$

where *S*_t_ is the total sliding force of the strip. *T*_t_ is the total anti-sliding force of the strip.

The equivalent safety factor of the 3D slope can be expressed as follow.

13 $$ F_{3} = \frac{{\sum\nolimits_{i = 1}^{n} {T_{{{\text{t,}}i}} } }}{{\sum\nolimits_{i = 1}^{n} {S_{{{\text{t,}}i}} } }} $$

where *n* is the number of bands divided by the sliding body.

Combined to Eqs. (12) and (13), the following expression can be written.

14 $$ F_{3} = \frac{{\sum\nolimits_{i = 1}^{n} {F_{2} S_{{{\text{t,}}i}} } }}{{\sum\nolimits_{i = 1}^{n} {S_{{{\text{t,}}i}} } }} $$

The difference of the obtained safety factor from Eq. (14) and the safety factor of real 3D slope depends on the sliding area. Therefore, the correction coefficient (d*s*/d*x*) of sliding area is introduced to modify Eq. (14) as follow.

15 $$ F_{3} = \frac{{\sum\nolimits_{i = 1}^{n} {F_{2} S_{{{\text{t,}}i}} \left( {\frac{{{\text{d}}s}}{{{\text{d}}x}}} \right)_{i} } }}{{\sum\nolimits_{i = 1}^{n} {S_{{{\text{t,}}i}} } }} $$

The safety factor of the strip section (*F*_2_) is obtained by adopting double wedges method and then the surface sliding force (*S*_t,*i*_) can be obtained through Eq. (2). Subsequently, the safety factor of quasi-3D slope can be obtained by summing the safety factor of each strip section by using Eq. (15). The overall procedure of this method can be summerized as follow: (1) Assuming the 3D sliding surface position and direction, (2) Dividing the sliding body into strips along the sliding direction, (3) Defining the locaiton and morphology of each strip section, (4) Calculating the safety factor of strip section (*F*_2_) and sliding force (*S*_t,*i*_) based on double wedges method, (5) Obtaining the quasi-3D safety factor of slope by using Eq. (15).

## Numerical simulation

### Engineering background and geological conditions

**Figure 3:**
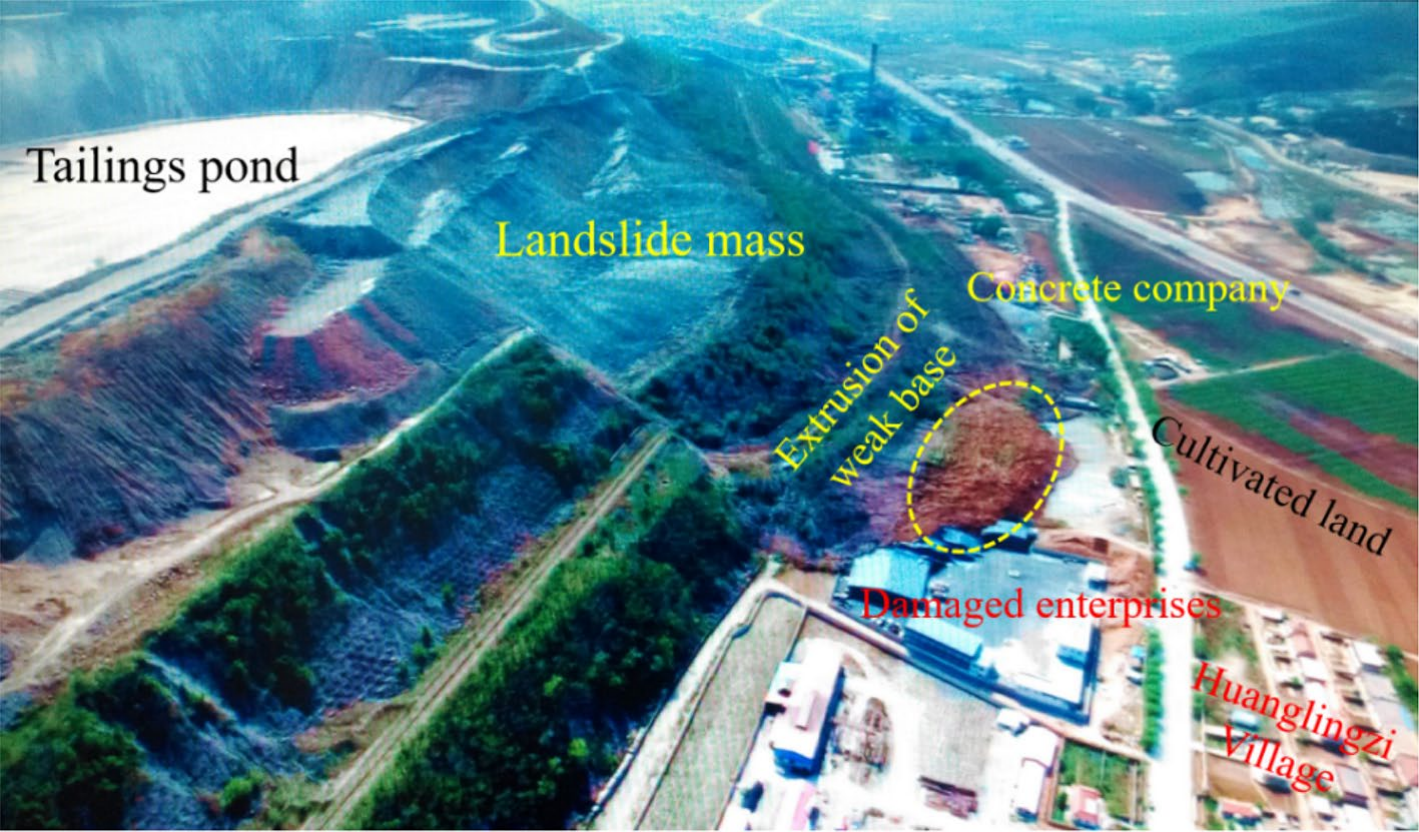
Overall landform of dump landslide area.

Taking the waste dump slope of an open-pit iron mine as the numerical simulation engineering background, the concrete company ground at the front edge of the slope toe of the waste dump once swelled, and a large landslide occurred at the north side, with a length of 550 m, a width of 890 m, and a volume of 7 million m^3^. It is a large landslide, as shown in Fig. 3.

**Figure 4:**
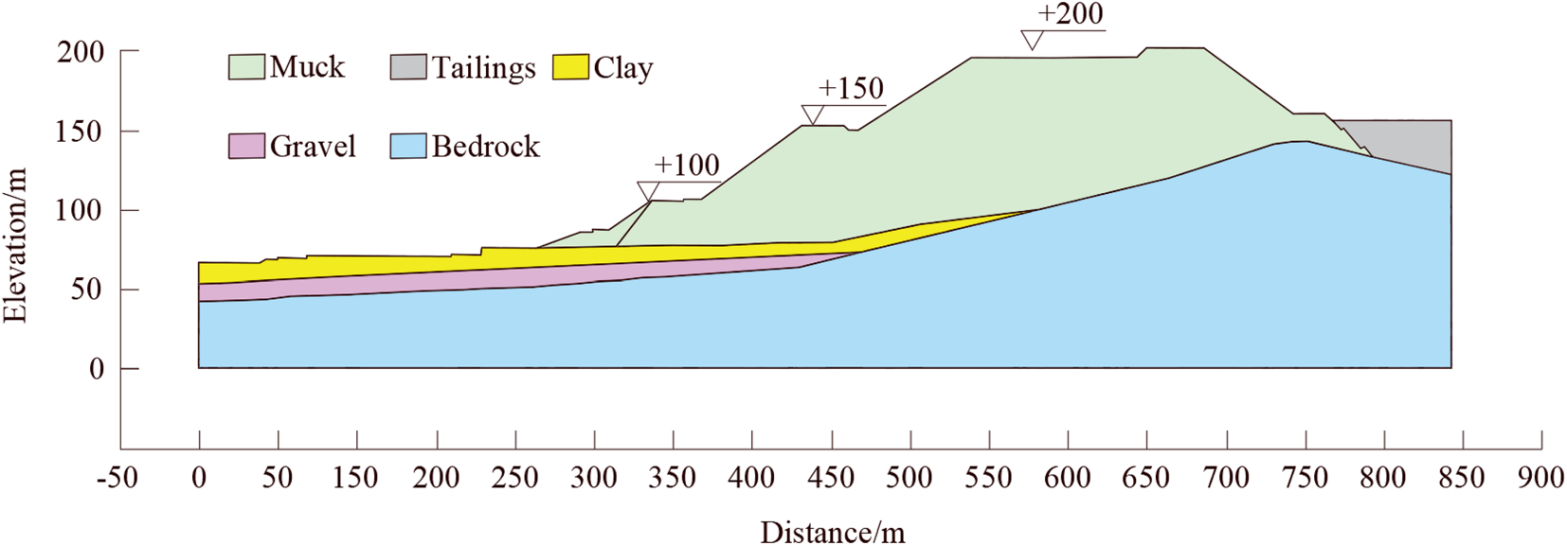
Typical geological profile of the main sliding zone.

Figure 4 illustrates a typical geological section of landslide area and the highest elevation and maximum difference of waste dump are 201.2 m and 130.6 m, respectively. The exposed strata in this area mainly consists of residue soil, slope deposit gravel, silty clay, pebble and sandstone. The muck is the main stratum of the waste dump with a bulk density of 2000 kg/m^3^, internal friction angle of 35° and cohesion of 1 kPa. The average thickness of silty clay is 9.34 m with a bulk density of 1900 kg/m^3^, internal friction angle of 8° and cohesion of 40 kPa. The average thickness of pebble layer is 13.59 m. The basement of the dump showed a trend of high in the south and low in the north, with the highest value of 143.4 m on the south side. The front of the basement is slow and the back is steep, and the maximum slope of the rear is 13°.

### Model establishment and result analysis

FLAC (Fast Lagangian Analysis of Continua) 3D is a continuum mechanics analysis software developed by Itasca, which has been widely used in the world. The software provides a concise operation interface, using the command flow to complete the establishment of the model and analysis and calculation. There are the following advantages in the algorithm: (1) The mixed discrete method is used to simulate the plastic flow and failure of materials more reasonably and accurately. (2) Regardless of the simulation of static or dynamic problems, the dynamic equation is used to solve the calculation, which eliminates the numerical obstacles in the simulation of physical processes. (3) The differential equation is solved by the explicit difference method.

**Figure 5:**
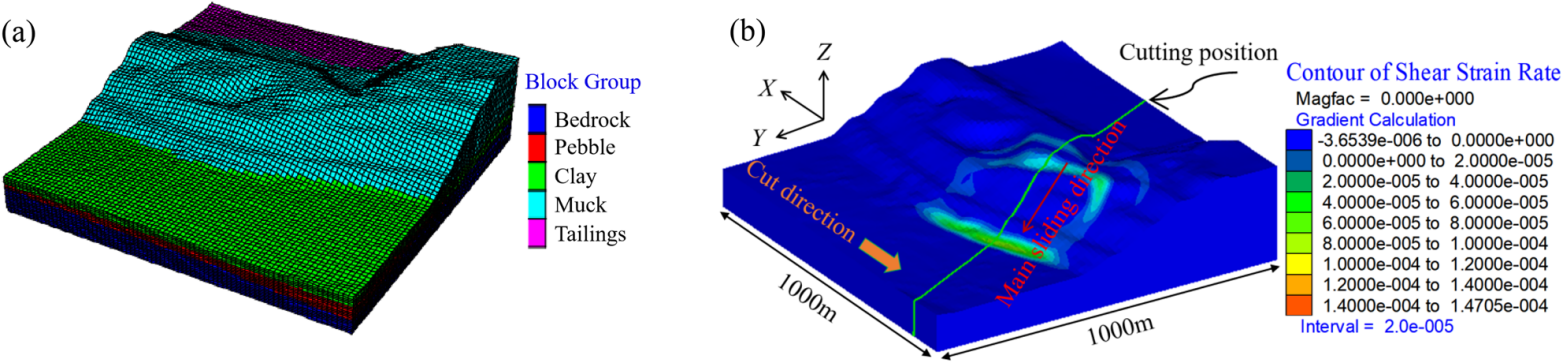
Full three-dimensional numerical model and shear strain rate of waste-dump slope.

The three-dimensional model of waste dump slope is established through FLAC3D software, as shown in Fig. 5a. The length and width of the calculation model are both 1000 m, covering the landslide area and the surrounding dump slope. The regional strata include bedrock layer, round gravel layer, clay layer, waste slag layer and tailings. The model consists of 128,000 nodes and 118,579 hexahedral eight-node units. When calculating, the bottom of the model is completely constrained, and the boundary on both sides is only horizontally constrained. According to the geological conditions and geological survey data, the physical and mechanical parameters of each stratum are shown in Table 1.

**Table 1:** Properties of soil and rock mass.

Material	Muck	Clay	Gravel	Bedrock	Tailings
Density* ρ* (kg/m^3^)	2000	1900	2000	2500	2300
Shear modulus *G* (Pa)	5.0E+06	2.8E+07	4.0E+06	7.0E+09	4.0 E+06
Bulk modulus *K* (Pa)	4.0E+06	1.1E+07	5.0E+06	2.0E+10	5.0 E+06
Cohesion *c* (Pa)	1.0E+03	4.0E+04	5.0E+03	3.0E+06	1.0E+02
Friction angle *φ* (°)	35	8	33	40	25
Tensile strength *R* (Pa)	0	0	5.0E+5	1.5E+6	0

**Figure 6:**
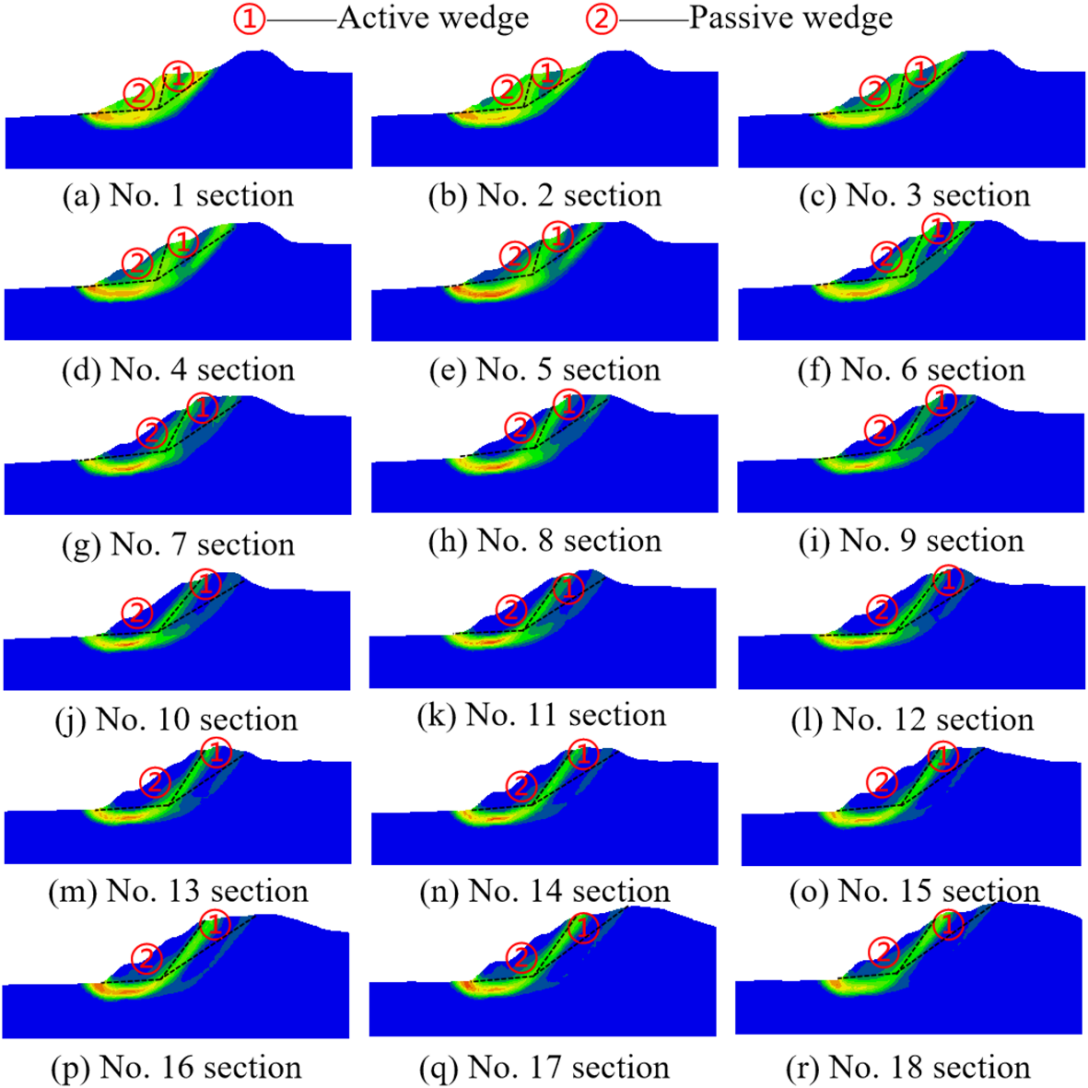
Contour of shear strain rate of vertical sections.

The Mohr Coulomb model is used to simulate and analyze the geotechnical materials, the strength reduction method is used to calculate the safety factor of the slope under the action of gravity field, the shear strain rate is used to describe the potential sliding surface, and the sliding failure of waste dump is inversely analyzed, as shown in Fig. 5b. The results show that the inverse safety factor of waste dump slope is 0.82, and the volume of the sliding body is 6.43 million m^3^ with the main sliding direction of 20° south by east. The landslide area is divided into 18 strips along the sliding direction and Fig. 6 illustrates the section of each strip. The larger shear strain rate in the shear strain rate nephogram of all sections is similar to the letter “*y*”, which is called “*y*” type distribution, and it is easy to form “*y*” type sliding surface. In addition, the “*y*” type potential sliding surface divides the sliding body into two blocks and the waste dump slope occurs double wedges failure along with the upper active wedge pushing the lower passive wedge to move horizontally. This is consistent with the results of bottom friction experiments based on typical geological Sects. ^12^.

**Figure 7:**
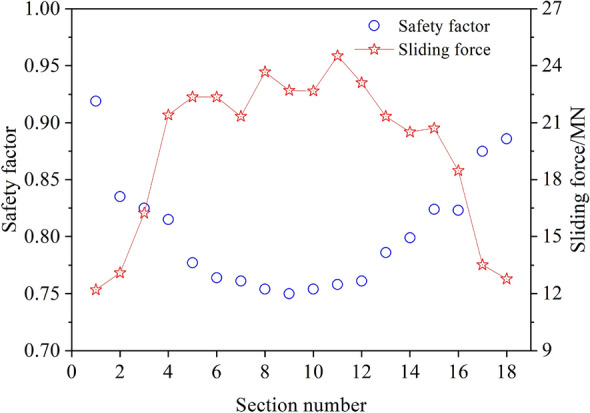
Safety factor and sliding force of 18 vertical sections.

It can be shown that the geometric shapes of waste dump slope at different section is different and the most dangerous sliding surface is located in the middle of the sliding body. Figure 7 illustrates the safety factor and sliding force of 18 sections. It shows that the safety factor of sliding surface obtained from double wedges method is in the range of 0.76–0.92, and the safety factor of section is larger in the closer boundary location of sliding body. Moreover, the correction coefficient (d*s*/d*x*) can be selected as 1–1.11 because it is larger at the edge of the sliding body and smaller at the middle of the sliding body. According to Eq. (15), the quasi-3D safety factor of slope is 0.817, which is basically consistent with the slope safety factor calculated by strength reduction method.

## Conclusion

In terms of waste dump slope with weak foundation, the limit equilibrium analysis is performed on double wedges and then the quasi-3D stability analysis method is proposed in this study by considering the influence of the geometry and sliding direction of slope surface to calculate the safety factor of waste dump slope. Some conclusions are obtained as follow.

- (1) In double wedges analysis method, the sliding body is divided into active wedge and passive wedge. Subsequently, the safety factor of waste dump slope and polygonal sliding surface are obtained by considering the interaction force and direction of double wedges.
  - (2) A quasi-three-dimensional double wedge stability calculation method for dump slope with weak base is proposed, which divides the sliding body into strips along the sliding direction. Using the safety factor of the middle section of the strip instead of the safety factor of the strip to correct the projection area, the safety factor of the three-dimensional slope can be obtained, and the error of the calculation result of the strength reduction method is 10%.
    - (3) Taking a waste dump slope as engineering background, the “*y*” type potential sliding surface divides the sliding body into two blocks and the waste dump slope occurs double wedges failure along with the upper active wedge pushing the lower passive wedge to move horizontally. The quasi-3D double wedges analysis method can be used as a simple and fast analysis method to evaluate the 3D slope stability in engineering practice.

## Data Availability

All data, models, and code generated or used during the study appear in the published article.

## Abbreviations

I: 1 Or 2, represent active wedge and passive wedge respectively
Li: Length of the potential sliding surface in the corresponding wedge
c, ci: Cohesion of rock and soil or the potential sliding surface in the corresponding wedge
φ, φi: Friction anlge of rock and soil or the potential sliding surface in the corresponding wedge
F: Safety factor of double wedges
αi: Angle between tensional sliding surface BD or shear sliding plane AB and horizontal plane
Ni: Normal tangential forces acting on the active wedge by the rear sliding surface
Ti: Normal tangential forces acting on the passive wedge by the basement
Wi: Gravity of each wedge
δ: Angle between the force P and the horizontal plane
Sm: Sliding force of the interface of double wedges
Nm: Vertical force of the interface of double wedges
L3: Length of the potential sliding surface BM
St: Total sliding force of the strip
Tt: Total anti-sliding force of the strip
n: Number of bands divided by the sliding body
ρ: Density of rock and soil
G: Shear modulus
K: Bulk modulus
R: Tensile strength
